# Editorial: Prokaryotic microbes in arid regions: distribution, environmental adaptation, biogeochemical cycling, and cultivation

**DOI:** 10.3389/fmicb.2024.1528610

**Published:** 2024-12-17

**Authors:** Zhen-dong Yang, Shuang Wang, Shaimaa Reda Hatab, Yong-Hong Liu

**Affiliations:** ^1^School of Architecture and Civil Engineering, Chengdu University, Chengdu, Sichuan, China; ^2^Heilongjiang Academy of Black Soil Conservation and Utilization, Harbin, China; ^3^Faculty of Organic Agriculture, Heliopolis University, Cairo, Egypt; ^4^State Key Laboratory of Desert and Oasis Ecology, Key Laboratory of Ecological Safety and Sustainable Development in Arid Lands, Xinjiang Institute of Ecology and Geography, Chinese Academy of Sciences, Urumqi, China

**Keywords:** arid regions, cultivation, prokaryotic microbes, environmental adaptation, biogeochemical cycling

## Introduction

The climatic conditions of arid regions are unique, offering challenging circumstances for microbial communities. Several physiological and metabolic strategies have allowed prokaryotic microbes (bacteria and archaea) to survive in these harsh environments. Their distribution, environmental adaptations, and ecological roles are essential to understanding several critical aspects, including their contributions to biogeochemical cycling (such as nitrogen fixation and sulfur metabolism), their potential applications in sustainable agriculture (through soil fertility and disease resistance enhancement), and their pivotal roles in maintaining ecosystem resilience under changing climate scenarios in arid regions. Where microbial biodiversity has declined with increasing aridity, studies have also shown that some taxa are exceptionally resilient and adaptable. According to Zhu et al., microbial communities differ among elevations in the eastern Pamirs, suggesting that elevation and environmental factors determine microbial diversity. The findings are extremely significant for understanding the succession of bacterial microorganisms in the plateau region, exploring the genetic resources of radiation-tolerant bacteria, and advancing the utilization of their biofunctional properties.

Similarly, Wang et al. investigated the barley farmland in the Qinghai-Xizang Plateau, and agricultural practices resulted in soil bacterial community homogenization and a possible diminishment of soil health and ecosystem functioning. Prokaryotic microbes evolved in arid regions and have developed different adaptation strategies to survive in the most extreme conditions. These adaptations include self-protective mechanisms such as synthesizing extracellular polysaccharides, entering a dormant state during drought, and utilizing alternate metabolic pathways to endure adverse conditions during desiccation. The endophytic actinobacterium *Streptomyces albidoflavus* produced secondary metabolites, enhancing plant growth and biocontrol of fungi. The ability to produce bioactive compounds that benefit their host provides these bacteria with an additional means to help these microbes survive while also making them better able to cope with environmental stresses such as drought, salinity, and extreme temperatures in arid regions.

Prokaryotic microorganisms are essential in biogeochemical cycles, particularly in nutrient-deficient and arid ecosystems. They are necessary for nitrogen fixation, organic matter decomposition, and carbon cycling. The study of Barkol Saline Lake (Liu et al.) showed the microbiological community response to changes in lake salinity level and nutrient availability related to sulfur metabolism and other biogeochemical processes. Understanding these microbial processes is essential for maintaining the health and fertility of soils that underlie agricultural systems that rely on the practices. There is strong evidence that climate-driven ecological changes and human activities caused the hypersaline lake Barkol Lake in northeast Xinjiang of China to develop alkaline soils and altered sediment characteristics. Advanced amplicon sequencing has been applied to studying microbial diversity in degraded areas, and it has been indicated that it is significantly lower in degraded areas than in lake sediments, with *Pseudomonadota* being the dominant group. Moreover, we found that while *Desulfobacterota* and *Bacillota* declined in degraded regions, the abundance of *Pseudomonadota, Acidobacteriota*, and *Actinobacteriota* increased. Deterministic processes drive the assembly of the microbial community, and the loss of structural complexity in degraded areas is predicted to lead to a substantially reduced sulfur metabolism.

The above work has implications for microbial adaptation in these and other highly saline ecosystems experiencing environmental change. Gu et al. investigated microbial diversity in a river lake continuum and factors that control microbial community structure. Sediment bacterial and fungal communities were found to be distinctive in Dongting Lake and its tributary rivers, with pH and macrozoobenthos being key drivers of community composition (Gu et al.). Random forest analysis was used in the research to predict community structures, emphasizing the need to understand the dynamics of these freshwater ecosystems. According to our findings, the species replacement processes dominated compositional dissimilarities between different sections of the river lake system. As a basis for conservation efforts and water resource management in arid regions, the importance of environmental factors in shaping microbial communities is stressed (Gu et al.).

Arid areas are unsuitable for agricultural production because of nutrient-poor soils and lack of water. However, prokaryotic microbes' integration into agricultural practices has been beneficial for increasing crop levels of resilience and productiveness. As a case in point, Zhao et al., describing how soil bacterial communities can increase a plant's resistance to Tobacco mosaic virus infection, show that certain bacterial strains can be exploited to enhance phyto-disease resistance. For example, using microbial inoculants (such as *Streptomyces albidoflavus*) promotes soil health and makes agricultural practices more sustainable by reducing the need for chemical fertilizers and pesticides. An endophytic actinobacterium, *S. albidoflavus*, isolated in the arid regions of Xinjiang, China, was analyzed using metabolomics technology to elucidate the mechanisms behind its antifungal and plant growth-promoting properties (Abdelshafy Mohamad et al.). The researchers identified 3,840 metabolites, among which 137 showed significant differences in abundance when the subjects bacteria were exposed to various fungal pathogens. The interaction between *S. albidoflavus* and fungal pathogens is complex, leading to the upregulation of 61 metabolites and the downregulation of 75 metabolites. The Principal Component Analysis (PCA) results showed that the first two components explained 43.64% of the variance, indicating that the metabolic profiles were distinct under different conditions (Abdelshafy Mohamad et al.). Several key metabolites are highlighted as possible antifungals (e.g., Tetrangulol, 4-Hydroxybenzaldehyde, and Cyclohexane), which possess antagonistic solid activity against fungal phytopathogens. It also showcases the impact of secondary metabolites on promoting plant growth, as crude extracts from *S. albidoflavus* enhanced the root and shoot length of plants tested. The findings of this study provide a rationale to use microbial metabolites in sustainable agriculture, especially in arid zones where traditional farming techniques might not be as effective (Abdelshafy Mohamad et al.).

The management of pathogens in crops cultivated in arid regions is of utmost significance. Weng et al. perform a comprehensive bibliometric analysis of potato disease research, showing a characterization of the field of potato disease research and clear trends and research gaps. It looked at publications from 2014 to 2023 and found 2,095 articles published in 103 countries. Leading contributors were the United States, China, and India; the United States alone contributed 23.01% of the total publications (Weng et al.). The study emphasizes the growing need to understand the critical role of prokaryotic microbes in suppressing potato diseases, especially in raising global food insecurity. Between 2018 and 2022, there was a surprising rise in publications, particularly as global food supply pressures ramp up. The analysis categorized research into three main areas: understanding disease mechanisms, advancing resistance breeding, and assessing disease severity and economic impacts.

The results highlight the necessity of a broader interdisciplinary perspective combining microbial ecology with plant pathology to improve crop disease resistance (Weng et al.). Zhao et al. studied the sequence of bacterial soil communities in Tobacco mosaic virus (TMV) infected *Nicotiana benthamiana*. Post-TMV infection, the study found significant changes in bacteria diversity and community structure, with beneficial bacteria like *Streptomyces* and *Sphingomonas* becoming more predominant under blue light exposure (Zhao et al.). The research provides insight into the complex interplay between pathogens and microbial populations and notes that initial bacterial community structure may affect a plant's susceptibility to viral infection. Their results suggested that manipulating soil microbial communities could facilitate the induction of plant resistance to pathogens, suggesting means to develop sustainable disease management strategies in agriculture (Zhao et al.).

The diversity and distribution pattern of microorganisms in arid areas are location-specific. Zhu et al. examined the bacterial community composition and distribution of bacteria in the eastern Pamir mountains. The soil samples were used in the study and analyzed by high-throughput sequencing, which found that the communities varied over the elevation and environmental factors (Zhu et al.). Community composition was found to vary according to microhabitat conditions, with *Actinobacteria* being the most abundant phylum. The findings also provide a picture of microbial biogeography in extreme environments and demonstrate the resilience of prokaryotic microbes to extreme conditions. This research highlights the ecological roles of these microorganisms in nutrient cycling and functioning in arid regions (Zhu et al.). Liu et al. further examined the responses of microbial diversity and function during Barkol Saline Lake degradation. Microbial community diversity was markedly lower in areas of dried-out former lake sediment compared to undisturbed sediment samples, suggesting significant ecosystem degradation (Liu et al.). It found *Pseudomonadota* to be the most dominant of the groups, with functional predictions indicating higher rates of sulfur metabolism in sediment samples than in degraded areas. The results demonstrate the sensitivity of these microbial communities to environmental transitions and thus emphasize the need for a deeper understanding of microbial dynamics in saline lake ecosystems to clarify effective approaches for conservation and management (Liu et al.). Zhang et al. used phyllosphere microbial communities in *Alhagi sparsifolia* across desert basins and seasons to explore diversity and dynamics. Co-occurrence network analysis examined microbial interactions, finding substantial seasonal and geographic differences in community composition (Zhang et al.). They also found that ecological factors such as leaf morphology and climate contributed to microbial diversity. The results highlight the involvement of phyllosphere microorganisms in Plant health and ecosystem functioning and can theoretically support restoration and resource management in arid ecosystems (Zhang et al.).

The climate conditions of Xizang and Xinjiang are similar. Wang et al. studied soil bacterial communities in barley farmland and wild systems on the Qinghai-Xizang Plateau. Agricultural practices turned out to be the reason for the uniformity of bacterial communities (which may influence soil health and ecosystem functioning). The research found that the phylum *Acidobacteria* was the most abundant in all ecosystems, and community composition was largely influenced by pH levels and organic matter content. The result indicates human activity's impact on microbial communities' diversity. It highlights the implication of harboring sustainable agricultural practices to sustainable microbial community management and soil resistance. For example, the study offers strategies such as crop rotation and reduced tillage to protect biodiversity and Ecosystem health (Wang et al.).

## Conclusion

This editorial reviews the studies highlighting the importance of prokaryotic microbes in arid regions, particularly regarding their distribution and environmental adaptation. At the same time, these studies increase our knowledge about microbial ecology and provide pointers for future sustainable agricultural practices to resolve the challenges of climate change and resource scarcity. Since the field of research evolves, it is necessary to incorporate microbial ecology with how we farm to ensure resilience and sustainability in arid environments. Combining the development of genomics and synthetic biology with computational modeling will enable us to better understand the interactions between prokaryotic microbes and their environments and to generate innovation to improve agricultural productivity and ecosystem health in some of the most challenging landscapes on the planet. These findings have a massive potential for application, from improving crop resilience to improving soil health to helping ensure more sustainable agricultural practices in arid regions.

